# Supplementary material for: Inner synchronization of controlled multi-valued logical networks
Source: PLoS One. 2026 Jan 27;21(1):e0340853. doi: 10.1371/journal.pone.0340853 (PMC12843602; doi:10.1371/journal.pone.0340853)
Supplement: S1 Table — This legend describes the table: the evolution process of node states under various initial states for the simulation examples of Theorem 2.1 and Theorem 2.2. (PDF) [file pone.0340853.s001.pdf]

Table of Node Values over Time

The supporting information is shown in the following 6 tables:

Table 1: Table of Node Values over Time

$t_0(x_1, x_2, x_3)$	$t_1(x_1, x_2, x_3)$	$t_2(x_1, x_2, x_3)$	$t_3(x_1, x_2, x_3)$
0,0,0	0,0,0	0,0,0	0,0,0
0,0,0.5	0,0.5,0	0,0,0	0,0,0
0,0,1	0,1,0	0,0,0	0,0,0
0,0.5,0	0,0,0	0,0,0	0,0,0
0,0.5,0.5	0,0.5,0	0,0,0	0,0,0
0,0.5,1	0,1,0	0,0,0	0,0,0
0,1,0	0,0,0	0,0,0	0,0,0
0,1,0.5	0,0.5,0	0,0,0	0,0,0
0,1,1	0,1,0	0,0,0	0,0,0
0.5,0,0	0,0,0	0,0,0	0,0,0
0.5,0,0.5	0,0.5,0	0,0,0	0,0,0
0.5,0,1	0,1,0	0,0,0	0,0,0
0.5,0.5,0	0,0,0	0,0,0	0,0,0
0.5,0.5,0.5	0,0.5,0	0,0,0	0,0,0
0.5,0.5,1	0,1,0	0,0,0	0,0,0
0.5,1,0	0,0,0	0,0,0	0,0,0
0.5,1,0.5	0,0.5,0	0,0,0	0,0,0
0.5,1,1	0,1,0	0,0,0	0,0,0
1,0,0	0,0,0	0,0,0	0,0,0
1,0,0.5	0,0.5,0	0,0,0	0,0,0
1,0,1	0,1,0	0,0,0	0,0,0
1,0.5,0	0,0,0	0,0,0	0,0,0
1,0.5,0.5	0,0.5,0	0,0,0	0,0,0
1,0.5,1	0,1,0	0,0,0	0,0,0
1,1,0	0,0,0	0,0,0	0,0,0
1,1,0.5	0,0.5,0	0,0,0	0,0,0
1,1,1	0,1,0	0,0,0	0,0,0

Table 2: Table of Node Values over Time

$t_0(x_1,x_2,x_3)$	$t_1(x_1,x_2,x_3)$	$t_2(x_1,x_2,x_3)$	$t_3(x_1,x_2,x_3)$	$t_4(x_1,x_2,x_3)$
0,0,0	0.5,0.5,0	0.5,0.5,0.5	0.5,0.5,0.5	0.5,0.5,0.5
0,0,0.5	1,0.5,0	0,0.5,0.5	0.5,0.5,0.5	0.5,0.5,0.5
0,0,1	0.5,1,0	0.5,0.5,0.5	0.5,0.5,0.5	0.5,0.5,0.5
0,0.5,0	0.5,0.5,0.5	0.5,0.5,0.5	0.5,0.5,0.5	0.5,0.5,0.5
0,0.5,0.5	0.5,0.5,0.5	0.5,0.5,0.5	0.5,0.5,0.5	0.5,0.5,0.5
0,0.5,1	0.5,1,0.5	0.5,0.5,0.5	0.5,0.5,0.5	0.5,0.5,0.5
0,1,0	0.5,0.5,0.5	0.5,0.5,0.5	0.5,0.5,0.5	0.5,0.5,0.5
0,1,0.5	0,0.5,0	0,0,0	0.5,0.5,0.5	0.5,0.5,0.5
0,1,1	0.5,1,0.5	0.5,0.5,0.5	0.5,0.5,0.5	0.5,0.5,0.5
0.5,0,0	0.5,0.5,0	0.5,0.5,0.5	0.5,0.5,0.5	0.5,0.5,0.5
0.5,0,0.5	0.5,0.5,0	0.5,0.5,0.5	0.5,0.5,0.5	0.5,0.5,0.5
0.5,0,1	0.5,1,0	0.5,0.5,0.5	0.5,0.5,0.5	0.5,0.5,0.5
0.5,0.5,0	0.5,0.5,0.5	0.5,0.5,0.5	0.5,0.5,0.5	0.5,0.5,0.5
0.5,0.5,0.5	0.5,0.5,0.5	0.5,0.5,0.5	0.5,0.5,0.5	0.5,0.5,0.5
0.5,0.5,1	0.5,1,0.5	0.5,0.5,0.5	0.5,0.5,0.5	0.5,0.5,0.5
0.5,1,0	0.5,0.5,0.5	0.5,0.5,0.5	0.5,0.5,0.5	0.5,0.5,0.5
0.5,1,0.5	0.5,0.5,0.5	0.5,0.5,0.5	0.5,0.5,0.5	0.5,0.5,0.5
0.5,1,1	0.5,1,0.5	0.5,0.5,0.5	0.5,0.5,0.5	0.5,0.5,0.5
1,0,0	0,0.5,0	0.5,0.5,0.5	0.5,0.5,0.5	0.5,0.5,0.5
1,0,0.5	0.5,0.5,0	0.5,0.5,0.5	0.5,0.5,0.5	0.5,0.5,0.5
1,0,1	0.5,1,0	0.5,0.5,0.5	0.5,0.5,0.5	0.5,0.5,0.5
1,0.5,0	0.5,0.5,0.5	0.5,0.5,0.5	0.5,0.5,0.5	0.5,0.5,0.5
1,0.5,0.5	0.5,0.5,0.5	0.5,0.5,0.5	0.5,0.5,0.5	0.5,0.5,0.5
1,0.5,1	0.5,1,0.5	0.5,0.5,0.5	0.5,0.5,0.5	0.5,0.5,0.5
1,1,0	0,0.5,0.5	0.5,0.5,0.5	0.5,0.5,0.5	0.5,0.5,0.5
1,1,0.5	0.5,0.5,0.5	0.5,0.5,0.5	0.5,0.5,0.5	0.5,0.5,0.5
1,1,1	0.5,1,0.5	0.5,0.5,0.5	0.5,0.5,0.5	0.5,0.5,0.5

Table 3: Table of Node Values over Time

$t_0(x_1,x_2,x_3)$	$t_1(x_1,x_2,x_3)$	$t_2(x_1,x_2,x_3)$	$t_3(x_1,x_2,x_3)$	$t_4(x_1,x_2,x_3)$
0,0,0	1,1,0	0,1,1	1,1,1	1,1,1
0,0,0.5	1,1,0	0,1,1	1,1,1	1,1,1
0,0,1	1,1,0	0,1,1	1,1,1	1,1,1
0,0.5,0	1,1,0.5	0.5,1,1	1,1,1	1,1,1
0,0.5,0.5	1,1,0.5	0.5,1,1	1,1,1	1,1,1
0,0.5,1	1,1,0.5	0.5,1,1	1,1,1	1,1,1
0,1,0	1,1,1	1,1,1	1,1,1	1,1,1
0,1,0.5	1,1,1	1,1,1	1,1,1	1,1,1
0,1,1	1,1,1	1,1,1	1,1,1	1,1,1
0.5,0,0	0.5,1,0	0.5,1,1	1,1,1	1,1,1
0.5,0,0.5	0.5,1,0	0.5,1,1	1,1,1	1,1,1
0.5,0,1	1,1,0	0,1,1	1,1,1	1,1,1
0.5,0.5,0	0.5,1,0.5	0.5,1,1	1,1,1	1,1,1
0.5,0.5,0.5	0.5,1,0.5	0.5,1,1	1,1,1	1,1,1
0.5,0.5,1	1,1,0.5	0.5,1,1	1,1,1	1,1,1
0.5,1,0	0.5,1,1	1,1,1	1,1,1	1,1,1
0.5,1,0.5	0.5,1,1	1,1,1	1,1,1	1,1,1
0.5,1,1	1,1,1	1,1,1	1,1,1	1,1,1
1,0,0	0,1,0	1,1,1	1,1,1	1,1,1
1,0,0.5	0.5,1,0	0.5,1,1	1,1,1	1,1,1
1,0,1	1,1,0	1,1,1	1,1,1	1,1,1
1,0.5,0	0,1,0.5	1,1,1	1,1,1	1,1,1
1,0.5,0.5	0.5,1,0.5	0.5,1,1	1,1,1	1,1,1
1,0.5,1	1,1,0.5	1,1,1	1,1,1	1,1,1
1,1,0	0,1,1	1,1,1	1,1,1	1,1,1
1,1,0.5	0.5,1,1	1,1,1	1,1,1	1,1,1
1,1,1	1,1,1	1,1,1	1,1,1	1,1,1

Table 4: Table of Node Values over Time

$t_0(x_1,x_2)$	$t_1(x_1,x_2)$	$t_2(x_1,x_2)$	$t_3(x_1,x_2)$
0,0	0,0	0,0	0,0
0,0.5	0,0	0,0	0,0
0,1	0,0	0,0	0,0
0.5,0	0,0.5	0,0	0,0
0.5,0.5	0.5,0.5	0.5,0.5	0.5,0.5
0.5,1	0.5,0.5	0.5,0.5	0.5,0.5
1,0	0,1	0,0	0,0
1,0.5	0.5,1	0.5,0.5	0.5,0.5
1,1	1,1	1,1	1,1

Table 5: Table of Node Values over Time

$t_0(x_1, x_2)$	$t_1(x_1, x_2)$	$t_2(x_1, x_2)$	$t_3(x_1, x_2)$
0,0	0,0	0,0	0,0
0,0.5	0,0	0,0	0,0
0,1	0,0	0,0	0,0
0.5,0	0,0.5	0,0	0,0
0.5,0.5	0.5,0.5	0.5,0.5	0.5,0.5
0.5,1	0.5,0.5	0.5,0.5	0.5,0.5
1,0	0,1	0,0	0,0
1,0.5	0.5,1	0.5,0.5	0.5,0.5
1,1	1,1	1,1	1,1

Table 6: Table of Node Values over Time

$t_0(x_1, x_2)$	$t_1(x_1, x_2)$	$t_2(x_1, x_2)$	$t_3(x_1, x_2)$
0,0	0,0	0,0	0,0
0,0.5	0,0	0,0	0,0
0,1	0,0	0,0	0,0
0.5,0	0,0.5	0,0	0,0
0.5,0.5	0.5,0.5	0,0	0,0
0.5,1	0,0.5	0,0	0,0
1,0	0,1	0,0	0,0
1,0.5	0,1	0,0	0,0
1,1	0,1	0,0	0,0
