# Supplementary material for: Inner synchronization of controlled multi-valued logical networks
Source: PLoS One. 2026 Jan 27;21(1):e0340853. doi: 10.1371/journal.pone.0340853 (PMC12843602; doi:10.1371/journal.pone.0340853)
Supplement: Attachment — Submitted filename: Response to Reviewers.pdf [file pone.0340853.s002.pdf]

Dear editor and reviewers,

We read your comments carefully. According to your advice, we made some modifications in the Revised Manuscript with Track Changes and the Manuscript, point by point as follows:

Reviewer #1: This paper is devoted to inner synchronization of controlled multi-valued logical networks. Some comments are supplied below to improve the quality.

TO Reviewer 1:

1. Please enrich Introduction with fault-tolerant optimized control of switched complex networks, synchronization of fractional reaction- diffusion complex networks to highlight the related topics.

Response: In the introduction, we have added content related to switched network optimization control and fractional order complex networks.

2. For (4), how about disturbances imposed on it?

Response: For equation (4), we discussed the issue of network disturbance in two different cases in the Revised Manuscript.

3. Are Definitions borrowed from existing results? If yes, please add theirs sources.

Response: NO. Under the driving and response mechanism, the synchronization definition in this article is the synchronization of a node driving a logical network, while existing synchronization problems related to logical networks are all the synchronization of one logical network driving another logical network.

4. The language is poor and there are many syntax errors.

Response: We have carefully reviewed the entire paper and have made comprehensive revisions to improve the grammar, syntax, and overall clarity. We believe the manuscript is now much clearer and easier to understand.

5. Please supply the comparison between the proposed theorems and the existing results on theory and simulation.

Response:

(1) Theoretical Comparisons: Our work is theoretically distinguished from prior art in the following aspects. While Reference [24] established an inner synchronization theorem for Boolean networks (binary-valued), and the study extended this to multi-valued logical networks, a key limitation remains: many such networks cannot achieve synchronization autonomously. Our primary theoretical contribution is to address this by formulating and solving the problem of achieving inner synchronization via a driving node, a control strategy that forces the network to synchronize.

(2) Simulation Comparisons :The simulation results demonstrate the effectiveness of our method:

The three-valued logical network (3) on Page 4 serves as a case, confirming that the network fails to achieve inner synchronization on its own. This is directly contrasted with the three-valued logical network (10) on Page 9, where the same network (3), when controlled by our proposed driving node, successfully achieves inner synchronization, with all nodes matching the driver's state.

This conclusion is further reinforced by Example 3.2 on Page 10, which shows another network transitioning from a non-synchronized state to a synchronized one upon the introduction of the driving node.

Reviewer #2: This paper investigates the inner synchronization of controlled multi-valued logical networks using the semi-tensor product approach. The topic seems to be interesting. However, the current manuscript suffers from significant language issues and a lack of sufficient comparison

with existing literature. These issues hinder the clarity and impact of the work. Major revisions are needed before this paper can be considered for publication.

TO Reviewer 2:

1. The English language usage in the paper needs significant improvement. There are some sentences with unclear or incorrect grammar and syntax, which makes it difficult for readers to understand the intended meaning. For example:

1) In Abstract: "...for the inner synchronization of multi-valued logical networks where the synchronization state is equal to the driving node state..." -> Suggest: "...for the case where the synchronized state of all network nodes equals the state of the driving node..."

2) Page 9: "Research on multi-valued logical networks has been widely applied to networked evolutionary games..." -> Suggest: "Multi-valued logical networks have been widely applied to the study of networked evolutionary games".

3) On the 5th line of Introduction: "...Based on the the above discovery..." -> Suggest: "...Based on the above discovery...".

4) In page 2, "Liu et al. [27] studies..." should be changed to "Liu et al. [27] studied...".

5) Semi-tensor product is abbreviated to STP in page 2, but not it is not used in page 3.

6) In Proposition 1.2, "When" should be changed to "when".

Response: We have carefully reviewed the entire paper and have made comprehensive revisions to improve the grammar, syntax, and overall clarity.

2. The proofs of Theorems 2.1 and 2.2 are hard to follow.

Response: In the process of theorems proof, it mainly involves STP operations, which are computationally complex. We have appropriately simplified the expressions involved in the theorems and added relevant propositions for the proof process.

3. The results of the paper should be compared with other works.

Response:

(1) Theoretical Comparisons: Our work is theoretically distinguished from prior art in the following aspects. While Reference [24] established an inner synchronization theorem for Boolean networks (binary-valued), and the study extended this to multi-valued logical networks, a key limitation remains: many such networks cannot achieve synchronization autonomously. Our primary theoretical contribution is to address this by formulating and solving the problem of achieving inner synchronization via a driving node, a control strategy that forces the network to synchronize.

(2) Simulation Comparisons :The simulation results demonstrate the effectiveness of our method:

The three-valued logical network (3) on Page 4 serves as a case, confirming that the network fails to achieve inner synchronization on its own. This is directly contrasted with the three-valued logical network (10) on Page 9, where the same network (3), when controlled by our proposed driving node, successfully achieves inner synchronization, with all nodes matching the driver's state.

This conclusion is further reinforced by Example 3.2 on Page 10, which shows another network transitioning from a non-synchronized state to a synchronized one upon the introduction of the driving node.

4. Can we studied the fixed-time synchronization of multi-valued logical networks? For fixed-time synchronization you can refer the works: 1) Applied Mathematics and Computation Volume 444, 2023, 127811 2) Journal of Applied Mathematics and Computing (2025) https://doi.org/10.1007/

s12190-025-02569-y.R2：

Response： It is possible to study the problem of fixed time synchronization in logical networks. Because the state of logical network nodes is finite, synchronization of logical networks is achieved in finite time. For Theorem 2.1 and Theorem 2.2, as long as there exists a specified a (an integer greater than zero) that satisfies Eq.(6) and Eq.(9), the conclusions of fixed time synchronization of logical networks can be obtained separately.

Thanks for your hard work. If the revision is not enough, please contact me, I'd like to follow your advice.

No.: PONE-D-25-32821

Manuscript entitled: Inner synchronization of controlled multi-valued logical networks

Authors: Yunyun Deng, Xiaolong Qi, Yi Liang
